# Brain 5‐lipoxygenase over‐expression worsens memory, synaptic integrity, and tau pathology in the P301S mice

**DOI:** 10.1111/acel.12695

**Published:** 2017-11-04

**Authors:** Alana N. Vagnozzi, Phillip F. Giannopoulos, Domenico Praticò

**Affiliations:** ^1^ Department of Pharmacology Center for Translational Medicine Lewis Katz School of Medicine Temple University Philadelphia PA 19140 USA

**Keywords:** 5‐Lipoxygenase, adeno‐associated virus, neuroinflammation, P301S, tauopathies, transgenic mice

## Abstract

Progressive accumulation of highly phosphorylated tau protein isoforms is the main feature of a group of neurodegenerative diseases collectively called tauopathies. Data from human and animal models of these diseases have shown that neuroinflammation often accompanies their pathogenesis. The 5‐lipoxygenase (5LO) is an enzyme widely expressed in the brain and a source of potent pro‐inflammatory mediators, while its pharmacological inhibition modulates the phenotype of a tau transgenic mouse model, the htau mice. By employing an adeno‐associated viral vector system to over‐express 5LO in the brain, we examined its contribution to the behavioral deficits and neuropathology in a different transgenic mouse model of tauopathy, the P301S mouse line. Compared with controls, 5LO‐targeted gene brain over‐expression in these mice resulted in a worsening of behavioral and motor deficits. Over‐expression of 5LO resulted in microglia and astrocyte activation and significant synaptic pathology, which was associated with a significant elevation of tau phosphorylation at specific epitopes, tau insoluble fraction, and activation of the cdk5 kinase. *In vitro* studies confirmed that 5LO directly modulates tau phosphorylation at the same epitopes via the cdk5 kinase pathway. These data demonstrate that 5LO plays a direct role in tau phosphorylation and is an active player in the development of the entire tau phenotype. They provide further support to the hypothesis that 5LO is a viable therapeutic target for the treatment and/or prevention of human tauopathy.

## Introduction

The hallmark pathological lesions in the Alzheimer's disease (AD) brain are composed of amyloid beta (Aβ) peptides and phosphorylated tau protein, which lead to formation and deposits of extracellular Aβ plaques and intracellular neurofibrillary tangles, respectively (Giannopoulos & Pratico, 2015a). Interestingly, the latter type of lesion also represents the main pathological signature of a large group of less investigated neurodegenerative diseases collectively called tauopathies. In general, human tauopathies, which among others include progressive supranuclear palsy (PSP), Pick's disease, and corticobasal degeneration, display progressive accumulation of hyperphosphorylated microtubule‐associated protein tau and tau pathology together with behavioral impairments and loss in synaptic integrity without any Aβ deposits (Spillantini & Goedert, 2013; Wang & Mandelkow, 2016).

Consistent data in the literature have reported exaggerated cellular and humoral inflammatory responses in the central nervous system (CNS) of both human and mouse models of tauopathy. However, the role and mechanisms of how neuroinflammation is linked to the pathogenesis of these diseases remain to be clarified (Yoshiyama *et al*., 2007; Mandrekar‐Colucci & Landreth, 2010; Laurent *et al*., 2017). 5‐lipoxygenase (5LO) is an enzyme that produces potent pro‐inflammatory lipid mediators, most of which are grouped under the name of leukotrienes (LTs) (Chinnici *et al*., 2007). In the CNS, this protein is highly expressed in the cortex and hippocampus and localizes to both neuronal and glial cells (Manev *et al*., 2000). Previously, we showed that 5LO acts as an endogenous modulator of Aβ formation *in vitro* and *in vivo* by influencing the development of the AD‐like phenotype in transgenic mouse models of the disease (Firuzi *et al*., 2008; Chu & Praticò, 2011a; Chu *et al*., 2012a; Giannopoulos *et al*., 2014). More recently, we reported that selective pharmacological inhibition of 5LO activity ameliorates tau neuropathology and behavioral impairments of htau mice, a transgenic mouse model expressing wild‐type human tau (Giannopoulos *et al*., 2015b).

In the current study, we evaluated the biological consequences of brain 5LO over‐expression in a different mouse model of human tauopathy, the P301S mice (Yoshiyama *et al*., 2007). This mouse line was chosen because mice develop severe pathology and behavioral deficits that closely resemble human tauopathy involving a mutation in the tau gene, as opposed to the htau mouse line which express regular human tau. As such, the P301S model is a good representative of human tauopathies with mutated human FTDP‐17. At the end of the study, we observed that brain 5LO over‐expression resulted in an exacerbation of their behavioral and motor deficits, a significant increase in tau phosphorylation and pathology, as well as disruption of synaptic integrity and neuroinflammation in these mice. The alteration in tau phosphorylation was secondary to a selective up‐regulation the cdk5 kinase pathway. *In vitro* data corroborated these findings showing that 5LO‐dependent tau phosphorylation was specifically dependent on the involvement of this kinase. Taken together, our data demonstrate a direct role of the 5LO pathway in modulating tau phosphorylation and neuropathology in a relevant mouse model of human tauopathy. They provide critical preclinical evidence to justify further testing of selective 5LO inhibitors as disease‐modifying agents for the treatment of AD and related tauopathies.

## Results

### 5LO gene transfer exacerbates behavior and motor deficits in P301S mice

**Figure 1:**
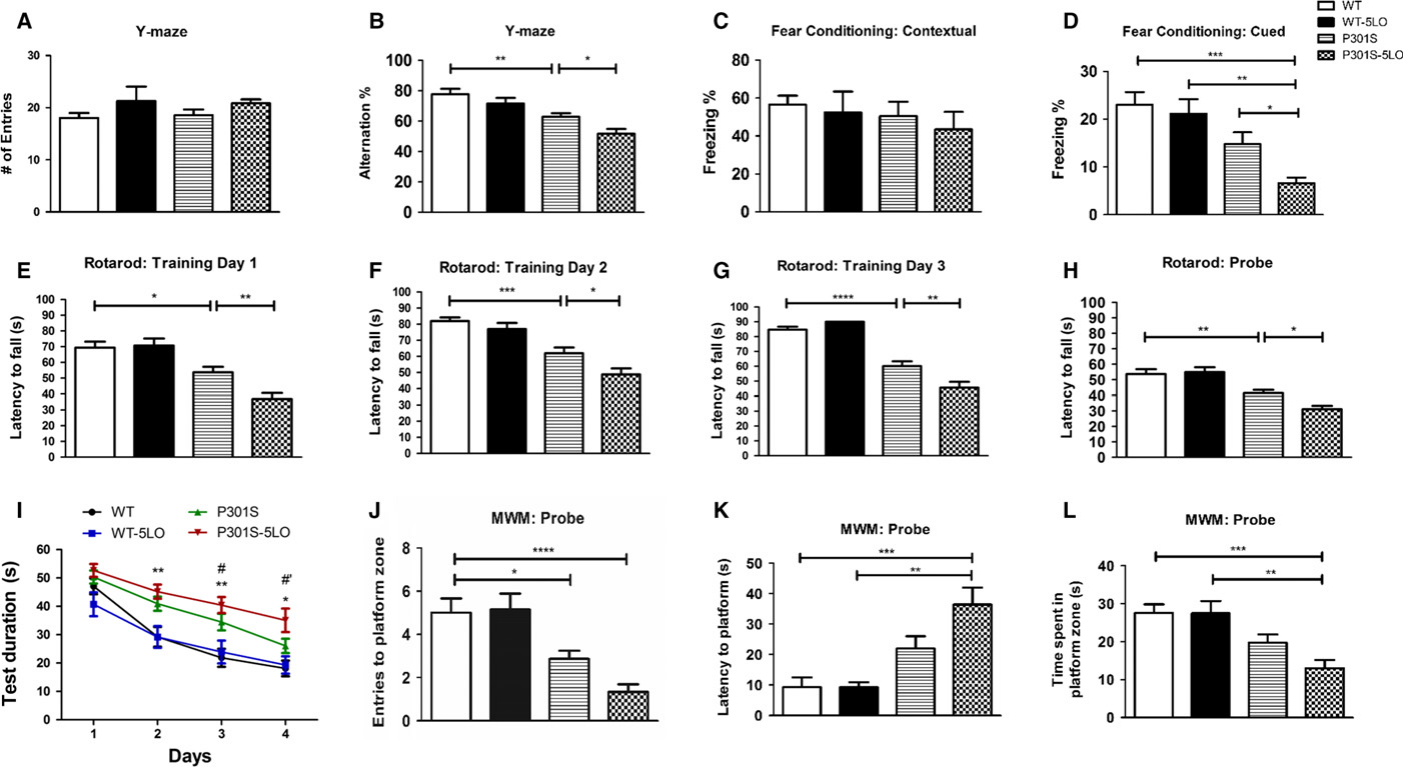
5LO gene over‐expression modulates behavior in P301S mice. (A) Total number of arm entries for WT, WT‐5LO, P301S, and P301S‐5LO mice at 9 months of age during the Y‐maze test (WT mean = 18.0, SEM = 1.0; WT 5LO‐AAV mean = 21.29, SEM = 2.775; P301S mean = 18.53, SEM = 1.154; P301S‐5LO AAV mean = 20.83, SEM = 0.767) (B) Percentage of alternations for each of the above group of mice (*P < 0.05, **P < 0.01) (WT mean = 77.71, SEM = 3.602; WT 5LO‐AAV mean = 71.51, SEM = 3.806; P301S mean = 62.9, SEM = 2.332; P301S‐5LO AAV mean = 51.74, SEM = 3.108). (C) Fear conditioning contextual phase for the four groups of mice (WT mean = 56.48, SEM = 4.803; WT 5LO‐AAV mean = 53.39, SEM = 11.11; P301S mean = 50.45, SEM = 7.57; P301S‐5LO AAV mean = 43.58, SEM = 9.111). (D) Fear conditioning cued phase for each of the above group of mice (*P < 0.05, **P < 0.01, ***P < 0.001) (WT mean = 23.06, SEM = 2.614; WT 5LO‐AAV mean = 21.13, SEM = 3.063; P301S mean = 14.79, SEM = 2.454; P301S‐5LO AAV mean = 6.617, SEM = 1.187). (E‐G) Rotarod training phase over three consecutive days (*P < 0.05, **P < 0.01, ***P < 0.001, ****P < 0.0001) (Day 1: WT mean = 69.38, SEM = 3.863; WT 5LO‐AAV mean = 70.74, SEM = 4.478; P301S mean = 53.73, SEM = 3.443; P301S‐5LO AAV mean = 37.00, SEM = 3.593. Day 2; WT mean = 81.94, SEM = 2.23; WT 5LO‐AAV mean = 76.97, SEM = 3.853; P301S mean = 61.93, SEM = 3.627; P301S‐5LO AAV mean = 48.82, SEM = 3.759. Day 3: WT mean = 84.70, SEM = 2.023; WT 5LO‐AAV mean = 90.00, SEM = 0.00; P301S mean = 60.11, SEM = 3.237; P301S‐5LO AAV mean = 45.61, SEM = 3.911). (H) Probe trial for the Rotarod (*P < 0.05, **P < 0.01) (WT mean = 53.61, SEM = 3.201; WT 5LO‐AAV mean = 54.84, SEM = 3.209; P301S mean = 41.35, SEM = 2.118; P301S‐5LO AAV mean = 31.09, SEM = 2.234). (I) Training phase of Morris water maze (MWM) as measured by latency to reach the platform zone over four consecutive days (*P < 0.05 P301S vs. WT; **P < 0.01 P301S vs. WT; #*P* < 0.01 P301S‐5LO vs. P301S; #’*P* < 0.001 P301S‐5LO vs. P301S). (J‐L) In the probe trial, we measure the number of entries to platform zone, latency to initial platform crossing, and time spent in platform zone for the four groups (*P < 0.05, **P < 0.01, ***P < 0.001, ****P < 0.0001 (J: WT mean = 5.00, SEM = 0.667; WT 5LO‐AAV mean = 5.143, SEM = 0.737; P301S mean = 2.867, SEM = 0.376; P301S‐5LO AAV mean = 1.333, SEM = 0.353. K: WT mean = 9.289, SEM = 3.184; WT 5LO‐AAV mean = 9.214, SEM = 1.661; P301S mean = 21.98, SEM = 4.036; P301S‐5LO AAV mean = 36.43, SEM = 5.548. L: WT mean = 27.63, SEM = 2.223; WT 5LO‐AAV mean = 27.47, SEM = 3.231; P301S mean = 19.68, SEM = 2.202; P301S‐5LO AAV mean = 13.00, SEM = 2.150). Values are expressed as mean ± SEM (n = 9 WT, 7 WT‐5LO, 15 P301S, and 12 P301S‐5LO).

To investigate the effect of 5LO gene transfer on cognition, mice were first tested on Y‐maze to assess working memory. Importantly, mice showed no differences in general locomotor activity as measured by the total number of arm entries (Fig. 1A). Conversely, P301S mice receiving AAV‐5LO (P301S‐5LO) had significantly lower number of arm alternations compared to P301S mice receiving empty vector (Fig. 1B). No significant differences were observed between WT groups receiving empty or AAV‐5LO vector (Fig. 1A,B). When mice underwent fear conditioning, no changes were seen between mice receiving empty vector or AAV‐5LO during the training phase (data not shown). While there were no significant differences observed when groups were tested on contextual recall, P301S mice showed significantly decreased freezing percentage during cued recall compared to WT counterparts, which was further reduced in the P301S‐5LO mice (Fig. 1C,D). Mice were also tested on the Rotarod to assess motor learning and ability. First, across groups, mice did not show baseline motor issues, as they spent comparable time on the rod during the training phase. However, P301S mice displayed greater motor learning deficits compared to WT counterparts throughout each training day, with AAV‐5LO treatment exacerbating these deficits (Fig. 1E–G). Similarly, during the probe test on Day 4, P301S‐5LO showed decreased motor function compared to both WT and P301S mice given empty vector (Fig. 1H). Lastly, mice were assessed on spatial learning via the Morris water maze paradigm. During the training phase over four consecutive days, P301S mice performed worse than their WT counterparts regarding time required to find the platform (Fig. 1I). Importantly, this deficit was exacerbated in mice treated with 5LO AAV on Days 3 and 4 when compared to P301S mice. During test day, as expected P301S mice had a lower number of entries and a reduced time in the platform zone, however, these aspects were exacerbated in the P301S‐5LO group (Fig. 1J,L). Additionally, P301S‐5LO mice took longer to find the platform than their WT counterparts as well as P301S control (Fig. 1K).

No significant differences between males and females were observed among the different groups as result of the 5LO over‐expression (data not shown).

### 5LO gene transfer results in 5LO over‐expression and neuroinflammation in P301S mice

**Figure 2:**
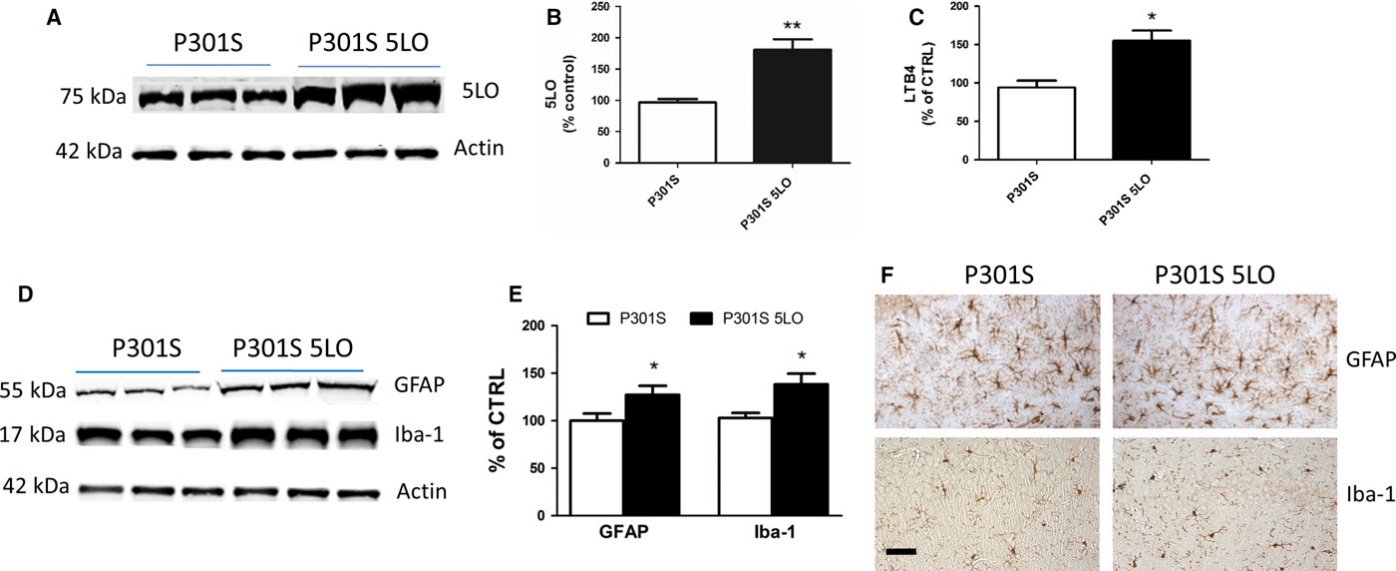
Brain over‐expression of 5LO modulates neuroinflammation in P301S mice. (A) Representative Western blot analyses for 5LO in brain cortex homogenates from P301S and P301S‐5LO mice. (B) Densitometric analyses of the immunoreactivities to the antibodies shown in panel A (**P < 0.01; n = 6 per group) (P301S mean = 100, SEM = 6.392; P301S‐5LO AAV mean = 169.15, SEM = 7.40). (C) LTB4 levels in brain cortex homogenates from P301S and P301S‐5LO mice (*P < 0.05; n = 5 per group) (P301S mean = 100, SEM = 9.131; P301S‐5LO AAV mean = 152.9, SEM = 13.58). (D) Representative Western blot analyses for glial fibrillary acidic protein (GFAP) and ionized calcium‐binding adapter molecule 1 (Iba‐1) in brain cortex homogenates from P301S and P301S‐5LO mice. (E) Densitometric analyses of the immunoreactivities shown in the previous panel (*P < 0.05; n = 4 per group) (P301S mean = 100, SEM = 7.625; P301S‐5LO AAV mean = 127.3, SEM = 9.383). Results are mean ± SEM. (F) Representative images of immunohistochemical staining in the hippocampus (HIPP) (CA1 region) for GFAP and Iba‐1 of P301S and P301S‐5LO mice (Scale bar = 50 μm).

Confirming the 5LO transgene expression in the brains of our P301S mice, we found significantly higher levels in 5LO protein level in P301S‐5LO compared with vector controls (Fig. 2A,B), as well as a 50% increase in 5LO activity as measured by leukotriene B4 (LTB4) levels in the brains of the same mice (Fig. 2C).

Compared with controls, P301S‐5LO mice had a significant increase in neuroinflammation as shown by the elevated steady‐state levels of the glial fibrillary acidic protein (GFAP), a marker of astrocyte activation, and ionized calcium‐binding adapter molecule 1 (Iba‐1), a marker of microglia activation (Fig. 2D,E). Immunohistochemical analyses confirmed the Western blot findings, showing an increase in immunoreactivity for GFAP and Iba‐1 protein (Fig. 2F).

### 5LO over‐expression modulates tau phosphorylation and pathology in P301S mice

**Figure 3:**
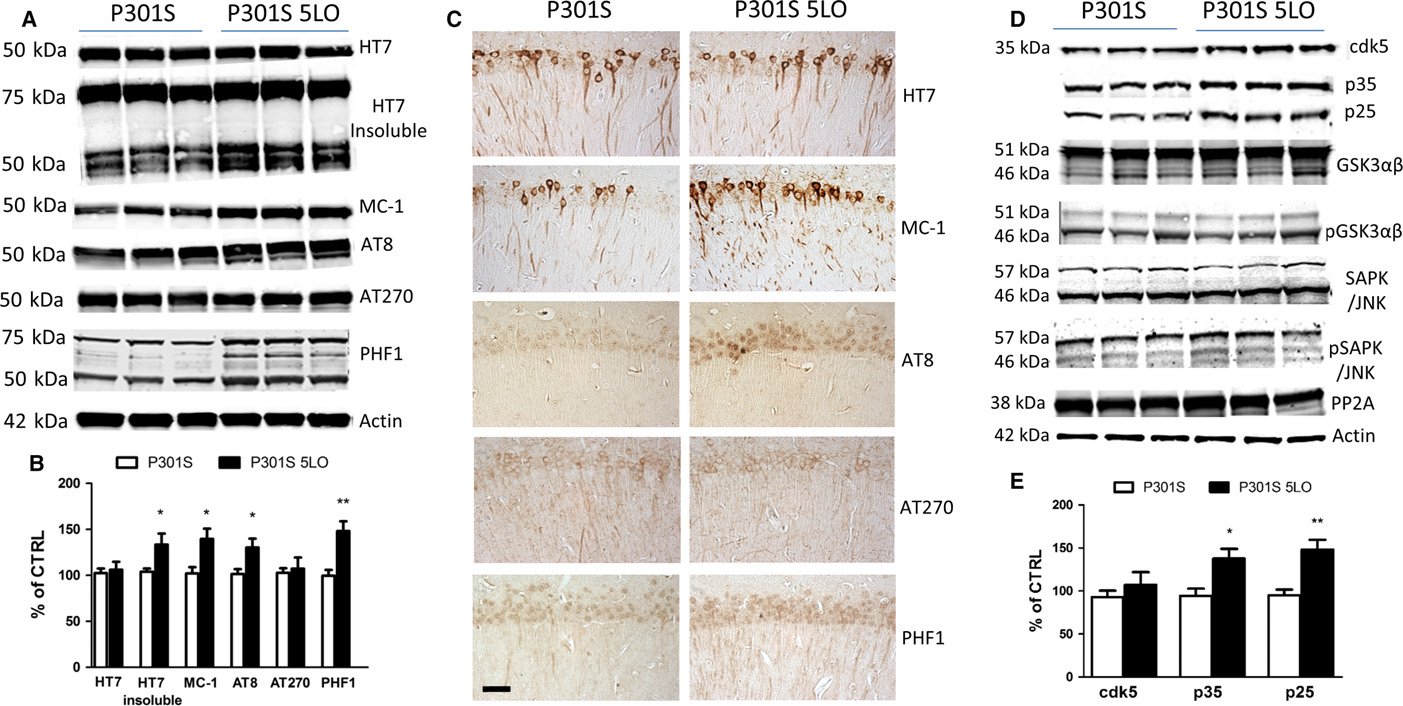
5LO regulates tau phosphorylation and metabolism in the brain of P301S mice. (A) Representative Western blot analyses for soluble tau and insoluble tau (HT7), MC1, and phosphorylated tau at residues S202/T205 (AT8), T181 (AT270), and S396/S404 (PHF1) in brain cortex homogenates from P301S and P301S‐5LO mice. (B) Densitometric analyses of the immunoreactivities to the antibodies shown in panel A (*P < 0.05, **P < 0.01; n = 5 per group) (HT7: P301S mean = 100, SEM = 4.928; P301S‐5LO AAV mean = 107.32, SEM = 8.784. HT7 insoluble; P301S mean = 100, SEM = 3.40; P301S‐5LO AAV mean = 133.45, SEM = 12.081. MC‐1: P301S mean = 100, SEM = 6.806; P301S‐5LO AAV mean = 139.57, SEM = 11.323. AT8: P301S mean = 100, SEM = 5.398; P301S‐5LO AAV mean = 130.21, SEM = 9.785. AT270: P301S mean = 100, SEM = 4.893; P301S‐5LO AAV mean = 107.29, SEM = 12.14. PHF1: P301S mean = 100, SEM = 6.492; P301S‐5LO AAV mean = 148.39, SEM = 10.821). (C) Representative images of immunohistochemical staining of the hippocampus (CA1 region) of P301S and P301S‐5LO mice for HT7, MC‐1, AT8, AT270, and PHF1 antibodies. (Scale bar = 50 μm). (D) Representative Western blot analyses for cyclin‐dependent kinase (cdk)5, p35, p25, glycogen synthase kinase (GSK3α, GSK3α, p‐GSK3α, p‐GSK3β), stress‐activated protein kinase/jun amino terminal kinase (SAPK/JNK1, SAPK/JNK2, p‐SAPK/JNK1, p‐SAPK/JNK2), and phosphatase protein‐2 (PP2)A protein levels in brain cortex homogenates from P301S and P301S‐5LO mice. (E) Densitometric analyses of the immunoreactivities to the antibodies from panel D (*P < 0.05, **P < 0.01; n = 5 per group. cdk5: P301S mean = 100, SEM = 7.604; P301S‐5LO AAV mean = 118.34, SEM = 12.812. p35: P301S mean = 100, SEM = 8.376; P301S‐5LO AAV mean = 137.82, SEM = 10.221. p25: P301S mean = 100, SEM = 6.544; P301S‐5LO AAV mean = 149.33, SEM = 11.22). Results are mean ± SEM.

Next, we assessed the effect of 5LO over‐expression on tau phosphorylation. To this end, we measured protein levels of total tau and its phosphorylated isoforms at different epitopes in brain cortex homogenates from P301S mice or P301S‐5LO. As shown in Fig. 3A, levels of total soluble tau, measured by HT7 antibody, remained unchanged when the two groups of mice were compared. However, P301S‐5LO showed an increase in phosphorylated tau at Ser202/Thr205 and Ser396/S404, as recognized by the antibodies AT8 and PHF1, respectively (Fig. 3A,B). Additionally, we found that compared to brains from P301S controls, the ones from P301S‐5LO mice had elevated levels of insoluble tau (Fig. 3A,B), as well as immunoreactivity to the antibody MC‐1 which, by recognizing N‐terminal amino acids 7–9 and C‐terminal amino acids 312–32 of the tau protein, is thought to detect changes in tau conformation. Confirming the Western blot data, histochemical staining showed greater phosphorylated tau epitopes and MC‐1 immunoreactivities in the brains of P301S‐5LO mice compared to P301S controls, while no changes were seen in total soluble tau as measured by HT7 antibody (Fig. 3C). No differences between males and females were observed among the different groups (data not shown).

To elucidate the potential mechanism that may be driving the changes in tau phosphorylation, we measured the levels of some key kinases that are responsible for phosphorylating tau. As shown in Fig. 3D, while we observed no significant changes in levels of cdk‐5 kinase, an increase in both of its known co‐activators, p35 and p25, was detected in P301S‐5LO brains when compared with P301S (Fig. 3D–E). By contrast, no differences between the two groups were found for the total or phosphorylated glycogen synthase kinase 3‐α (GSK3‐α) and GSK3‐β, total and phosphorylated stress‐activated protein kinase (SAPK/JNK), and protein phosphatase 2A (PP2A) (Fig. 3D).

### 5LO over‐expression affects synaptic integrity

**Figure 4:**
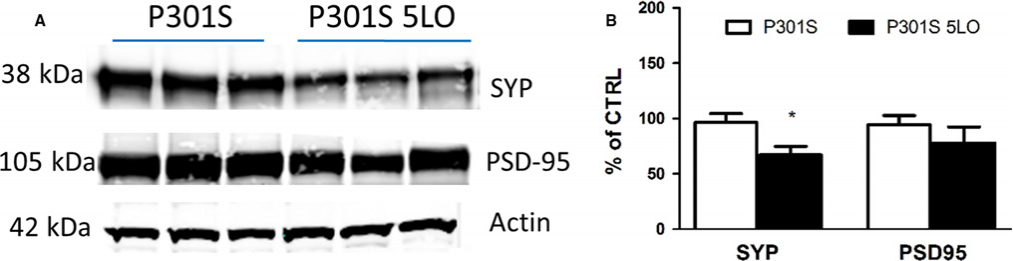
5LO over‐expression affects synaptic integrity in the P301S mice. (A) Representative Western blot analyses for synaptophysin (SYP) and postsynaptic density protein 95 (PSD95) in brain cortex homogenates from P301S and P301S‐5LO mice. (B) Densitometric analyses of the immunoreactivities shown in the previous panel (*P < 0.05; n = 4 per group. SYP: P301S mean = 100, SEM = 6.226; P301S‐5LO AAV mean = 69.172, SEM = 5.748. PSD‐95: P301S mean = 100, SEM = 8.061; P301S‐5LO AAV mean = 79.819, SEM = 13.63). Results are mean  ± SEM.

Cognitive deficits, as well as tau phosphorylation, are often associated with changes in synaptic integrity. Thus, we investigated whether biomarkers of this aspect of the synapse were affected by 5LO over‐expression in P301S mice. Compared to P301S mice, the mice over‐expressing 5LO manifested a significant decrease in the steady‐state levels of synaptophysin, a presynaptic marker (Fig. 4A,B). By contrast, no changes were observed between the two groups when measuring levels of postsynaptic density protein 95 (PSD‐95) (Fig. 4A,B).

No differences between males and females were observed among the different groups as result of the 5LO over‐expression (data not shown).

### 5LO increases tau phosphorylation via cdk5 kinase

**Figure 5:**
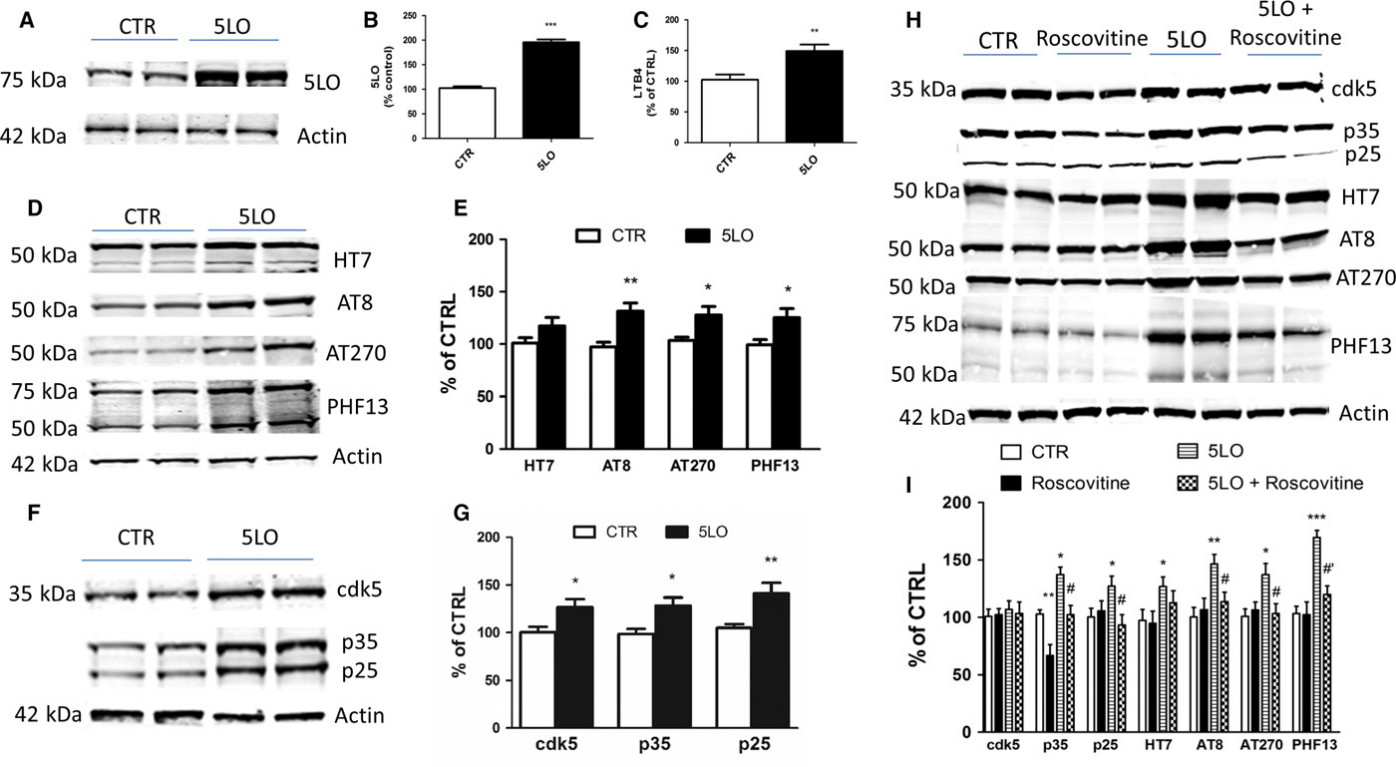
5LO modulates tau phosphorylation by a cdk5‐dependent mechanism. N2A neuronal cells stably expressing human tau protein were transiently transfected with empty vector or human 5LO pcDNA3.1 for 48 h, and then, the supernatants and cell lysates were harvested for biochemistry. (A) Representative Western blot analysis for 5LO protein in cells lysates transfected with 1 μg empty vector (CTR), or 5LO plasmid (5LO). (B) Densitometric analyses of the immunoreactivity to the antibody shown in previous panel (***P < 0.001; P301S mean = 100, SEM = 3.70; P301S‐5LO AAV mean = 195.83, SEM = 5.431). (C) Levels of LTB4 in conditioned media from the same cells described in the previous panel (**P < 0.01; P301S mean = 100, SEM = 7.374; P301S‐5LO AAV mean = 148.37, SEM = 9.538). (D) Representative Western blot analysis for total tau (HT7) and phosphorylated tau at residues S202/T205 (AT8), T181 (AT270), and S396 (PHF13) in lysates from the same cells. (E) Densitometric analyses of the immunoreactivities to the antibodies shown in panel D (*P < 0.05, ** *P* < 0.01; HT7: P301S mean = 100, SEM = 7.625; P301S‐5LO AAV mean = 127.3, SEM = 9.383. AT8: P301S mean = 100, SEM = 7.625; P301S‐5LO AAV mean = 127.3, SEM = 9.383. AT270: P301S mean = 100, SEM = 7.625; P301S‐5LO AAV mean = 127.3, SEM = 9.383. PHF13: P301S mean = 100, SEM = 7.625; P301S‐5LO AAV mean = 127.3, SEM = 9.383). (F) Representative Western blot analyses of cdk5, p35, and p25 protein levels in lysates from control (CTR) or 5LO transfected neuronal cells. (G) Densitometric analyses of the immunoreactivities to the antibodies shown in panel F (*P < 0.05 cdk5: P301S mean = 100, SEM = 5.677; P301S‐5LO AAV mean = 126.74, SEM = 8.684. p35: P301S mean = 100, SEM = 5.406; P301S‐5LO AAV mean = 130.53, SEM = 8.396. p25: P301S mean = 100, SEM = 4.152; P301S‐5LO AAV mean = 142.11, SEM = 10.042). Results are mean ± SEM (n = 2 per condition, three individual experiments). (H) Representative Western blots of cdk5, p35, p25, total tau (HT7), phosphorylated tau at residues S202/T205 (AT8), T181 (AT270), and S396 (PHF13) in lysates from cells transfected with 5LO or vector control (CTR) in the presence or absence of roscovitine (20 μm) for 24 h. (I) Densitometric analyses of the immunoreactivities to the antibodies shown in the previous panel (*P < 0.05 vs. control; **P < 0.01 vs. control; ***P < 0.001 vs. control; #*P* < 0.05 vs. 5‐LO; #’*P* < 0.01 vs. 5LO). Values represent mean ± SEM. p35: CTR mean = 100, SEM = 3.688, Roscov mean = 66.831, SEM = 9.478, 5LO mean = 137.35, SEM = 6.479, 5LO + Roscov mean = 102.31, SEM = 7.969. p25: CTR mean = 100, SEM = 7.675, Roscov mean = 105.73, SEM = 8.766, 5LO mean = 127.28, SEM = 8.758, 5LO + Roscov mean = 92.33, SEM = 9.171. HT7: CTR mean = 100, SEM = 9.419, Roscov mean = 94.86, SEM = 10.64, 5LO mean = 126.84, SEM = 8.327, 5LO + Roscov mean = 112.76, SEM = 10.653. AT8: CTR mean = 100, SEM = 8.394, Roscov mean = 106.76, SEM = 10.11, 5LO mean = 146.53, SEM = 8.212, 5LO + Roscov mean = 114.07, SEM = 8.309. AT270: CTR mean = 100, SEM = 6.356, Roscov mean = 102.54, SEM = 6.903, 5LO mean = 137.29, SEM = 9.799, 5LO + Roscov mean = 105.34, SEM = 8.647. PHF13: CTR mean = 100, SEM = 6.374, Roscov mean = 102.30, SEM = 10.15, 5LO mean = 170.76, SEM = 6.104, 5LO + Roscov mean = 116.03, SEM = 7.554 (n = 2 per condition, three individual experiments).

To corroborate our *in vivo* findings, N2A cells stably expressing human tau were transiently transfected with empty vector or 5LO pcDNA3.1 for 48 h. At this time point, compared with control cells, cells receiving 5LO plasmid had a significant increase in the steady‐state levels of 5LO protein, which was accompanied by a similar increase in its enzymatic activity, as shown by the higher levels of LTB4 in the supernatant (Fig. 5A–C). No significant changes were observed for the amount of total tau protein between the two groups. However, we observed that cells with 5LO over‐expression had a statistically significant increase in the phosphorylated tau isoforms at epitopes Ser202/Thr205, Thr181, and Ser396, as recognized by the antibodies AT8, AT270, and PHF13, respectively (Fig. 5D,E). Under this experimental condition, we observed that steady‐state levels of cdk5 as well as the levels of its co‐activators, p35 and p25, were significantly enhanced in the same cells (Fig. 5F,G). To further elucidate the interaction between 5LO and cdk5 on the observed effect on tau phosphorylation, we used a pharmacological approach. N2A cells stably expressing human tau were pretreated with a specific cdk5 inhibitor, roscovitine (20 μm), 24 h before 5LO over‐expression, and the same parameters as above were assessed (Zhang *et al*., 2004). This concentration and incubation time were chosen based on previous studies conducted by our group. Specifically, we found that this concentration and incubation time produced the most efficient effect of suppressing cdk5 activity (Chu *et al*., 2012b). As seen in Fig. 5H,I, we observed that roscovitine reduced the levels of p25/p35 in cells over‐expressing 5LO and this effect was associated with a significant reduction in phosphorylated tau as recognized by antibodies AT8, AT270, and PHF13.

## Discussion

In the present study, we show for the first time that brain over‐expression of 5LO causes a significant increase in tau phosphorylation, neuroinflammation, synaptic pathology, and behavioral impairments in the P301S mice, a transgenic mouse model of tauopathy. Taken together, these data establish that this enzymatic pathway is an active player in tauopathy pathogenesis *in vivo* and provide further critical support toward the research effort aimed at implementing and developing drugs that target this protein as novel therapeutic agents for this disease.

With the recent shift away from the amyloid hypothesis, research has focused on finding novel therapeutic approaches to alleviate both primary and secondary pathologies associated with AD and related tauopathies. Given the heterogeneous nature of tauopathies, dissecting the contribution of these secondary pathologies in disease progression, such as synaptic dysfunction and neuroinflammation, in addition to the signature accumulation of hyperphosphorylated tau deposits, remains highly relevant in the field of neurodegeneration (Arendt *et al*., 2016; Lamb *et al*., 2016). The 5LO enzymatic pathway, which upon activation produces potent inflammatory mediators, has recently emerged as a novel target in neurodegeneration (Chu & Praticò, 2011b; Joshi *et al*., 2014). Importantly, our group has shown that steady‐state levels of 5LO are increased in brain cortices from subjects with a postmortem diagnosis of PSP and that pharmacological inhibition of its activity ameliorates tau pathology and behavioral deficits in the htau mice (Giannopoulos *et al*., 2015b). To gather further support and confirm that 5LO is indeed an active player in the tauopathy phenotype, in the current study, we implemented a different mouse model of tauopathy, the P301S line, and a new and opposite experimental approach, 5LO gene over‐expression, which by stably increasing the levels and activity of this pathway in the CNS of these mice, should exacerbate their pathological phenotype.

First, we showed that 5LO over‐expression results in a worsening of their memory performance as assessed in the Y‐maze paradigm, which by recording spontaneous alternation behavior, measures working memory in rodents (Götz & Ittner, 2008). Compared with control, mice over‐expressing 5LO had a significant reduction in the percentage of alternations, which reflects their immediate working memory. By contrast, the same treatment did not alter the number of entries, which reflects the general motor activity of the animals. Additionally, P301S mice over‐expressing 5LO performed the worst compared to all other groups in freezing percentage when assessed in the cued phase of the fear conditioning paradigm. Interestingly, 5LO over‐expression in the P301S mice had a negative effect also on memory and spatial learning as assessed by the Morris water maze test. Thus, compared with tau mice controls, P301S‐5LO mice showed greater latency to find the platform zone, significantly lower number of entries into the same zone, and spent less time in the quadrant of the platform zone.

Interestingly, we observed changes in the Morris water maze (MWM), a hippocampal‐dependent cognitive test, but no changes in the contextual fear conditioning paradigm, another hippocampus‐dependent paradigm. Because of this apparent discrepancy, it is important to note that several other brain regions may also influence spatial learning. Thus, in addition to the hippocampus, thalamic structures, amygdala, prefrontal cortex, and cerebellum are among the different brain regions known to be involved when assessing performance in the MWM (Puzzo *et al*., 2014). Such involvement needs to be considered when evaluating cognitive performance, specifically in terms of region specificity of each behavioral paradigm.

Finally, mice were tested in their motor skills on the Rotarod. During the training phase and probe test, P301S mice performed worse than WT counterparts and showed reduced motor learning skills. However, P301S over‐expressing 5LO manifested a significant worsening of motor learning compared to P301S controls. No significant effect of 5LO gene over‐expression on any of the implemented behavioral paradigms was detected in the WT mice groups, suggesting a specific effect of 5LO on the transgene.

As learning and memory task responses are known to be directly modulated by synaptic function and integrity, next we assessed two well‐established synaptic markers, synaptophysin, and PSD‐95 (Hoover *et al*., 2010; Sydow *et al*., 2011). Compared with P301S controls, the same mice over‐expressing 5LO displayed decreased levels of the presynaptic marker synaptophysin, while no differences were seen in levels of PSD‐95, indicating that 5LO can influence synaptic integrity particularly at the presynaptic level.

Confirming the efficiency and long‐lasting biological effect of our gene transfer, we showed that compared with mice receiving empty vector, P301S‐5LO mice have a significant increase in the steady‐state levels of the 5LO protein. Importantly, this increase was associated with a corresponding elevation of its enzymatic activity, as demonstrated by the significant increase in the levels of its main metabolic product, the LTB4, in the same brain samples. In support of the pro‐inflammatory actions of the higher amount of LTB4 in the CNS of P301S‐5LO mice, we observed that indeed the neuroinflammatory response was also significantly affected. To this end, compared with controls, over‐expression of 5LO resulted in significantly higher levels of GFAP, a marker of astrocyte activation, and Iba1, a protein marker of microglia activation (Bellucci *et al*., 2004; McGeer & McGeer, 2010).

Coincidental with the increase in neuroinflammatory responses, we observed that 5LO over‐expression resulted in altered tau phosphorylation at specific tau epitopes. Interestingly, we also observed that while the 5LO gene over‐expression did not influence the levels of total soluble tau protein, it did alter the solubility and conformation of tau protein as shown by the higher amount of insoluble tau fraction and a significant increase in the immune reactivity to the MC‐1 antibody.

Aging in combination with a chronic inflammatory environment could enhance neuronal vulnerability to pathological insults such as tau accumulation. This highlights the importance of studying the 5LO pro‐inflammatory pathway, as this enzyme is not only upregulated centrally but also in the vasculature, thus upregulation of this pathway may perpetuate a cycle of inflammatory mediator activation. In our case, however, as our mice specifically over‐expressed 5LO in the CNS, we can rule out the peripheral contribution to the observed phenotype.

To elucidate potential mechanisms for the changes in tau phosphorylation, we assessed some of the kinases and phosphatases that are considered modulators of these post‐translational modifications of tau *in vivo*. Among them, we observed that compared with P301S control mice, brains from tau mice over‐expressing the 5LO had a significant increase in the cdk5 kinase pathway. In particular, while we did not detect any change in the total levels of the cdk5, *per se*, we found that levels of both of its co‐activators, p35 and p25, were significantly elevated in brains from P301S‐5LO mice, suggesting a role for the cdk5 pathway in the 5LO‐dependent effect on tau phosphorylation and neuropathology.

To further corroborate the role of 5LO in tau phosphorylation via the cdk5 kinase pathway, we implemented an *in vitro* approach congruent to our mouse model and characterized by neuronal cells stably expressing human tau protein, in which we up‐regulated 5LO. Compared to control cells, 5LO over‐expression resulted in a significant increase in tau phosphorylation, which was associated with higher levels of cdk5, and p35 and p25, its two co‐activators. The functional role of this kinase was confirmed using roscovitine, a specific pharmacological inhibitor of cdk5 activation (Zhang *et al*., 2004). Under our experimental conditions, we indeed observed that roscovitine was able to prevent the 5LO‐dependent increase in tau phosphorylation at the same epitopes. It is of interest to note that while in both *in vitro* and *in vivo* we observed a significant increase in the levels of the two main activators of the cdk5, namely p35 and p25, steady‐state levels of total cdk5 were significantly increased only in the *in vitro* studies. We speculate that this apparent discrepancy is secondary to the different sources implemented: brain tissue, which contains different types of cells, vs. the simple neuronal cells. In any event, it is important to consider that as monomeric cdk5 needs to associate with its activators to display its full function, our results are consistent with an increase in its activity both *in vivo* and *in vitro* (Liu *et al*., 2016).

In conclusion, our *in vivo* and *in vitro* data directly implicate the 5LO enzymatic pathway as a key regulator of neuropathology and the tauopathy behavioral phenotype in a mouse model of pure tauopathy, the P301S line, via a cdk5 pathway‐dependent mechanism. The elucidation of the role of this enzyme in tauopathy pathogenesis provides a strong biological support for the hypothesis that pharmacologic inhibition of 5LO represents a novel and viable therapeutic strategy for treating or halting the clinical and pathological symptoms of human tauopathies.

## Experimental procedures

### Construction and preparation of AAV2/1 vector

The construction, packaging, purification, and titering of the recombinant AAV2/1 vector expressing 5LO were performed as previously reported (Chu & Praticò, 2011a).

### Injection of AAV2/1 to neonatal mice

The P301S mice (PS19 line) expressing human mutant microtubule‐associated protein tau, *MAPT*, driven by the mouse prion protein (Prnp) promoter were used in this study (Yoshiyama *et al*. 2007). The injection procedures were performed as described previously (Chu & Praticò, 2011a). Briefly, two microliters of AAV2/1‐5LO (1.3 × 10^13^ genome particles mL^−1^) was bilaterally injected into the cerebral ventricle of newborn mice using a 5‐μL Hamilton syringe. A total of forty‐three pups were used for the study; nineteen were injected with AAV2/1‐5LO (7 wild‐type P301S−/−, 12 P301S+/−; Females = 10, Males = 9) and twenty‐four were injected with empty vector (9 wild‐type, 15 P301S Females = 13, Males = 11). All animals were housed on a 12‐h light/dark cycle in a pathogen‐free environment and given regular chow and water *ad libitum*. Animals were then followed until they were 8‐ to 9‐month‐old when they first underwent behavioral testing. Two weeks later, mice were sacrificed. Upon euthanasia, mice were perfused with ice‐cold 0.9% PBS containing EDTA (2 mmol/L), pH 7.4. Brains were removed, gently rinsed in cold 0.9% PBS, and immediately dissected into two halves. One‐half was stored at ‐80̊ C for biochemistry, while the other half was fixed in 4% paraformaldehyde diluted in PBS, pH 7.4, for immunohistochemical studies.

### Behavioral tests

All the animals were handled for at least 3–4 consecutive days before testing. They were tested in random order, and the experimenter conducting the tests was unaware of the genotype/treatment.

### Y‐maze

The Y‐maze apparatus consisted of three arms (32 cm long and 610 cm wide) with 26‐cm walls (San Diego Instruments, San Diego, CA, USA). Testing was always performed in the same room and at the same time to ensure environmental consistency as previously described (Di Meco *et al*., 2014; Li *et al*., 2014). Briefly, each mouse was placed in the center of the Y‐maze and allowed to explore freely during a 5‐min session as a measure of spontaneous alternating behavior. The sequence and total number of arms entered were video‐recorded. An entry into an arm was considered valid if all four paws entered the arm. An alternation was defined as three consecutive entries into three different arms (1, 2, 3, or 2, 3, 1, etc.). Percentage of alternation was calculated using the following formula: (total alternation number/total number of entries−2) × 100.

### Fear conditioning

Fear conditioning experiments were performed following methods previously described (Di Meco *et al*., 2014, 2017) . Tests were conducted in a conditioning chamber equipped with black methacrylate walls, transparent front door, a speaker, and grid floor (Start Fear System; Harvard Apparatus)

### Rotarod

Mice were tested on Rotarod as previously described (Lauretti *et al*., 2017). Briefly, a Rotarod instrument with automatic timers and falling sensors (Omnitech Electronics, Columbus, OH, USA) was used and testing was performed on four consecutive days. The mice were placed individually on a 30‐mm‐diameter rotating cylinder suspended above a cage floor. The length of time the mice managed to remain on the rod was automatically recorded. The mice underwent six trials per day, and the maximal observation time for each trial was 90 s. During the training phase (day 1–3), the speed of the rotation was increased gradually from 0 to 15 r.p.m. during the first 15 s and held constant at that rate for the rest of the trial (75 s). During the test (day 4), the speed of rotation was accelerated gradually from 0 to 90 r.p.m. during the 90 s of the trial.

### Morris water maze

To perform the Morris water maze (MWM), we used a white circular plastic tank (122 cm in diameter, walls 76 cm high), filled with water maintained at 22°±2°C, and made opaque by the addition of a nontoxic white paint, as previously described (Di Meco *et al*., 2014; Li *et al*., 2014). Mice were trained for four consecutive days to find a Plexiglas platform submerged in water from four different starting points. If they failed to find the platform within 60 s, they were manually guided to the platform and allowed to remain there for 15 s. Mice were trained to reach a training criterion of 20 s (escape latency). Mice were assessed in the probe trial, which consisted of a free swim lasting for 60 s without the platform, 24 h after the last training session. Animals’ performances were monitored using Any‐Maze™ Video Tracking System (Stoelting Co., Wood Dale, IL, USA) which provided data for the acquisition parameters (latency to find the platform and distance swam and) and the probe trial parameters (number of entries in the target platform zone of the platform and time in quadrants).

### Western blot analyses

RIPA extracts from mouse brain homogenates were used for Western Blot analyses as previously described (Di Meco *et al*., 2017; Lauretti *et al*., 2017). Briefly, samples were electrophoresed on 10% Bis‐Tris gels or 3–8% Tris‐acetate gel (Bio‐Rad, Richmond, CA, USA), transferred onto nitrocellulose membranes (Bio‐Rad), and then incubated overnight at 4 ° C with the appropriate primary antibodies; anti‐5LO [dilution: 1:200] (Santa Cruz, Dallas, TX, USA), anti‐HT7 [1:200] (Thermo, Waltham, MA, USA), anti‐MC‐1 [1:50] (Dr. Peter Davies), anti‐AT8 [1:100] (Thermo), anti‐AT270 [1:200] (Thermo), anti‐PHF1 [1:100] (Santa Cruz), anti‐PHF13 [1:100] (Thermo), anti‐SYP [1:300] (Santa Cruz), anti‐PSD95 [1:200] (Thermo), anti‐GSK3α/β [1:100] (Cell Signaling, Danvers, MA, USA), anti‐pGSK3α/β [1:100] (Cell Signaling), anti‐SAPK/JNK [1:100] (Cell Signaling), anti‐pSAPKJNK [1:100] (Cell Signaling), anti‐cdk5 [1:200] (Santa Cruz), anti‐p35/p25 [1:100] (Santa Cruz), anti‐PP2A [1:200] (Santa Cruz), anti‐GFAP (Santa Cruz), anti‐Iba1 [1:100] (Thermo), and anti‐Beta actin [1:500] (Santa Cruz). After three washings with T‐TBS (pH7.4), membranes were incubated with IRDye 800CW‐labeled secondary antibodies (LI‐COR Bioscience, Lincoln, NE, USA) at room temperature for 1 h. Signals were developed with Odyssey Infrared Imaging Systems (LI‐COR Bioscience, Lincoln, Nebraska). β‐Actin was always used as internal loading control.

### Sarkosyl insolubility assay

The assay for insoluble tau was performed as previously described (Li *et al*., 2014). Briefly, ultracentrifugation and sarkosyl extraction (30 min in 1% sarkosyl) was used to obtain soluble and insoluble fractions of tau from brain homogenates. Insoluble fractions were washed one time with 1% sarkosyl, then immunoblotted with the HT7 antibody.

### Immunohistochemistry

Immunostaining was performed as previously described (Di Meco *et al*., 2017; Lauretti *et al*., 2017). Briefly, serial coronal sections were mounted on 3‐aminopropyl triethoxysilane (APES)‐coated slides. Every eighth section from the habenular to the posterior commissure (8–10 sections per animal) was examined with unbiased stereological principles. The sections used for testing HT7, MC‐1, AT8, AT270, PHF1, GFAP, and Iba1 were deparaffinized, hydrated, rinsed with phosphate‐buffered saline, and pretreated with citric acid (10 mm) for 5 min for antigen retrieval, then with 3% H_2_O_2_ in methanol for 30 min to eliminate endogenous peroxidase activity and with blocking solution (2% normal serum in Tris buffer, pH 7.6). The sections were incubated with appropriate primary antibody overnight at 4°C then with secondary antibody at room temperature and developed using the avidin–biotin complex method (Vector Laboratories, Burlingame, CA, USA) with 3,3‐diaminobenzidine (DAB) as chromogen.

### LTB_4_ assay

RIPA extracts from mouse brain homogenates from P301S‐empty and their age‐matched P301S‐5LO AAV counterparts were assayed for LTB_4_ levels using a specific LTB_4_ ELISA kit (Enzo Life Sciences, Farmingdale, NY, USA), and following the instructions of the manufacturer.

### Cell line and treatment

Neuro‐2 A neuroblastoma (N2A) cells stably expressing human tau (N2A‐tau) were cultured in Dulbecco's modified Eagle medium supplemented with 10% fetal bovine serum, 100 U mL^−1^ streptomycin (Mediatech, Herdon, VA, USA), and 100 mg mL^−1^ Hygromycin (Invitrogen, Carlsbad, CA, USA) at 37°C in the presence of 5% CO_2_. The cells were cultured to 70% confluence in six‐well plates and then transfected with 1 μg empty vector or human 5LO pcDNA3.1 (Dr. Colin Funk, Queen's University, Kingston, Canada) using Lipofectamine reagent (Invitrogen) according to the manufacturer's instructions and as previously described (Chu *et al*., 2012b). After 48‐h treatment, supernatants were collected and cell harvested in lytic buffer for biochemistry analyses.

### Statistical analysis

All the data are expressed as mean ± SEM. The one‐way analysis of variance test, Bonferroni multiple‐comparison test, and the two‐tailed Student's t‐test were performed using Prism 5.0 (Graph Pad Software, La Jolla, CA, USA) to determine the statistical significance, with significance set at *P *<* *0.05.

## Funding

This study was supported in part by grants from the Alzheimer Art Quilt Initiative, and the Wanda Simone Endowment Fund for Neuroscience (to D.P).

## Author contributions

A.V., P.F.G., and D.P. designed the study, developed the experimental design, performed data analyses, and wrote the study. A.V. and P.F.G. performed the experiments. All authors discussed the results and commented on the manuscript.

## Conflict of interests

The authors have no conflicting financial interest to disclose.
